# Supplementary material for: PGC-1β maintains mitochondrial metabolism and restrains inflammatory gene expression
Source: Sci Rep. 2022 Sep 26;12:16028. doi: 10.1038/s41598-022-20215-6 (PMC9512823; doi:10.1038/s41598-022-20215-6)
Supplement: Supplementary file 1 — Supplementary Figures. [file 41598_2022_20215_MOESM1_ESM.docx]

PGC-1β maintains mitochondrial metabolism and restrains inflammatory gene expression

Hannah Guak^1^, Ryan D. Sheldon^2^, Ian Beddows^3^, Alexandra Vander Ark^1^, Matthew J. Weiland^1^, Hui Shen^3^, Russell G. Jones^1^, Julie St-Pierre^4^, Eric H. Ma^1^, and *Connie M. Krawczyk^1^

^1^Department of Metabolism and Nutritional Programming, Van Andel Research Institute, Grand Rapids, MI 49503, USA

^2^Metabolomics and Bioenergetics Core, Van Andel Research Institute, Grand Rapids, MI 49503, USA

^3^Department of Epigenetics, Van Andel Research Institute, Grand Rapids, MI 49503, USA

^4^Department of Biochemistry, Microbiology and Immunology, University of Ottawa, Ottawa, ON K1N 6N5, Canada

* Corresponding author

# Supplementary Information

Figure S1 – Gene expression of *Ppargc1b* with LPS or IFN-β treatment

Bone marrow-derived DCs were left unstimulated or stimulated with LPS (100 ng/mL) or IFN-β (1000 U/mL) for 18 hours. Fold changes of *Ppargc1b* expression were made relative to *Hprt*. Data are from one experiment representative of three experiments (mean and s.e.m. of triplicates).


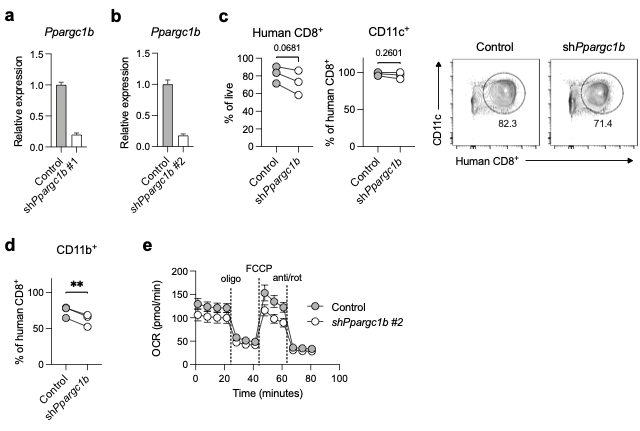


Figure S2 – Knockdown of *Ppargc1b* by shRNA

DCs were transduced with control shRNA or two different *Ppargc1b* shRNA (hairpin #1 in **(a)** and hairpin #2 in **(b)**), and knockdown was confirmed by RT-qPCR. **(c)** Proportion of live cells expressing human CD8 (marker of transduced cells; left), proportion of transduced cells expressing CD11c (middle), representative plot showing human CD8^+^ CD11c^+^ cells gated on live cells (right). **(d)** Proportion of transduced cells expressing CD11b. **(e)** OCR was measured over time of DCs transduced with control shRNA or *Ppargc1b* shRNA #2 with sequential treatments of oligomycin (oligo), FCCP, and antimycin/rotenone (anti/rot). Data are of one experiment representative of **(a)** at least three experiments (mean and s.e.m. of three replicates), **(b)** two experiments (mean and s.e.m. of three replicates), or **(e)** three experiments (mean and s.d. of six replicates). Data from **(c,d)** are of three experiments pooled together, with each individual experiment represented by a circle and values from the same experiment joined by a line. Statistical significance in **(c,d)** was determined by paired *t-*test. ** p < 0.01

Figure S3 – Activation marker expression at steady state and with IFN-β treatment in PGC-1β-deficient DCs

DCs were transduced with control shRNA or *Ppargc1b* shRNA and **(a)** intracellular levels of cytokine IL-12p40, **(b)** geometric MFI of CCR7, and **(c)** intracellular levels of IL-6 were determined by flow cytometry. **(a)** Histogram (left) and IL-12p40^+^ cells expressed as a percentage (right) of transduced CD11c^+^ DCs treated with IFN-β. **(c)** Histogram (left column) and IL-6 geometric MFI (right column) of transduced CD11c^+^ cells left untreated (top row) or treated with IFN-β (bottom row) for 4 hours. Data are of one experiment representative of two experiments (mean and s.d. of duplicates). Data from **(b)** are of three experiments pooled together, with each individual experiment represented by a circle and values from the same experiment joined by a line. Statistical significance was determined by paired *t*-test.

Figure S4 – PGC-1β deficiency increases autophagy and does not affect apoptosis

DCs were transduced with control or *Ppargc1b* shRNA and **(a)** examined for apoptosis, represented by the frequency of cells that were positive for both ApoTracker Green and eFluor 506 Fixable Viability Dye. DCs were cultured in 0, 1 or 10 mM glucose as indicated for 6 hours, and **(b)** gene expression of *Sqstm1* relative to *Hprt* was measured*.* 50 μM chloroquine (CQ) was added for the last 2 hours of culture and **(c)** SQSTM1 was visualized by western blot (left) and relative intensity of SQSTM1 bands to β-actin determined by densitometric analysis (right). **(d)** LC3-I/II visualized by western blot (left) and relative intensity of LC3-II to β-actin (middle). Autophagic flux was calculated by determining the difference between the intensity of LC3-II normalized by β-actin in the presence and absence of chloroquine (right). Data are of one experiment representative of **(a)** one experiment (mean and s.d. of triplicates), **(b-d)** three experiments. Statistical significance in **(b)** was determined by two-way ANOVA. * p < 0.05, *** p < 0.001

Figure S5 – Metabolite analysis following ^13^C-glucose pulse, IL-1β production and NLRP3 inflammasome activation, and gene expression analysis of PGC-1β-deficient DCs

**(a)** Relative abundance of labeled (^13^C) intracellular lactate (m+3 isotopomer) from DCs transduced with control or *Ppargc1b* shRNA and cultured with U-[^13^C]-glucose for 6 h prior to metabolite extraction. **(b)** IL-1β production by transduced DCs activated with LPS (100 ng/mL) overnight or 3 hours of LPS followed by addition of aluminium hydroxide (alum; 250 μg/mL) overnight. **(c)** Western blot of NLRP3, ASC, pro-IL-1β and cleaved IL-1β of transduced DCs left untreated or treated with LPS (100 ng/mL) or IFN-β (1000 U/mL) for 3 hours followed by addition of alum overnight to the indicated conditions. **(d)** Select Reactome pathways that are significantly enriched in sh*Ppargc1b* versus control DCs. **(e,f)** Heat maps showing the expression of genes involved in **(e)** respiratory electron transport (in unstimulated and IFN-β-treated conditions), **(f)** TCA cycle and cristae formation (IFN-β-treated). **(g)** Plot of data significance (-log_10_(p)) versus log_2_(fold change) for changes in expression of genes encoding proteins involved in mitochondrial fusion (*Opa1* and *Mfn2*) and fission (*Dnm1l*) in *shPpargc1b* DCs compared to control DCs. Data in **(e)** show unstimulated control versus unstimulated sh*Ppargc1b* DCs and IFN-β-treated control versus IFN-β-treated sh*Ppargc1b* DCs as separate comparisons. Data in **(a-c)** are of one experiment representative of two experiments (mean and **(a)** s.e.m. of triplicates or **(b)** s.d. of triplicates). ** p < 0.01

Figure S6 – Changes in expression of genes encoding inflammatory factors

RNA sequencing was performed on DCs transduced with control or *Ppargc1b* shRNA and left unstimulated or stimulated with IFN-β (1000 U/mL) for 6 hours. Heat maps show the expression of genes involved in signaling by interleukins (left) and MAPK family signaling cascades (right). Data show unstimulated control versus unstimulated sh*Ppargc1b* DCs and IFN-β-treated control versus IFN-β-treated sh*Ppargc1b* DCs as separate comparisons within each pathway.

Figure S7 – Uncropped blots

Uncropped blots of **(a)** p-AMPK, AMPK, and β-actin corresponding to Fig. 3g, **(b)** OXPHOS cocktail (ETC complexes I-V) and VDAC corresponding to Fig. 4b, **(c)** SQSTM1 and β-actin corresponding to Supplementary Fig. S4c, **(d)** β-actin and LC3-I/II corresponding to Supplementary Fig. S4d, and **(e)** NLRP3, pro-IL-1β, cleaved IL-1β, ASC, and α-tubulin corresponding to Supplementary Fig. S5c.
